# Sensors for Indoor Mapping and Navigation

**DOI:** 10.3390/s16050655

**Published:** 2016-05-09

**Authors:** Kourosh Khoshelham, Sisi Zlatanova

**Affiliations:** 1 Department of Infrastructure Engineering, University of Melbourne, Parkville 3010 VIC, Australia; 2 3D Geoinformation, Faculty of Architecture and the Built Environment, Delft University of Technology, Delft, P.O.Box 5043, 2600 GA, The Netherlands; S.Zlatanova@tudelft.nl

With the growth of cities and increased urban population there is a growing demand for spatial information of large indoor environments. This includes location information of pedestrians, goods and robots in indoor spaces as well as detailed indoor maps and models, which can be used for route planning, navigation guidance and several other applications.

Obtaining spatial information in indoor environments is a challenge. In the absence of GNSS signals, a range of technologies have been used for positioning and mapping indoors. However, limitations in accuracy and coverage, dependence on infrastructure and calibration issues limit the existing solutions to only a few application scenarios.

Articles in this special issue provide an overview of the most recent developments in sensor technology and methodology for indoor positioning, mapping and new applications enabled by indoor location information. The majority of these works focus on the positioning problem. Inertial sensors, accelerometers and gyroscopes, which are available on most smartphones, are used for positioning in [1,2,3,4,5,6]. The position estimation in these works is mainly based on the Pedestrian Dead Reckoning (PDR) approach, which involves step length estimation using accelerometer data. In [7,8,9,10,11,12] inertial sensors are combined with map information or landmarks to strengthen the positioning solution. In [13] inertial sensors are integrated with a 2D laser scanner to obtain the position of a mobile robot. The simultaneous map generation and positioning methods in [14,15] combine inertial sensors with camera images. In [16] accelerometer data and ambient radio sensed by a smartphone are used to detect place visits of the user. The pressure sensor built in smartphones (usually referred to as barometer) is used to estimate the vertical position or to recognize vertical movements in [7,10,13,17,18].

The magnetic sensor built in smartphones (usually referred to as magnetometer or digital compass) is used for attitude estimation as part of a positioning system in [1,4,7,8,9,10,12,19,20]. In [21] a complete magnetic positioning system is described, in which the positioning is based on a magnetic sensor measuring the strength of the magnetic field generated by a number of coils installed in the environment.

Indoor positioning using WiFi signals is investigated in [22,23,24,25,26,27]. The common approach to position estimation in these works is based on the received signal strength (RSS) and the fingerprinting method. In [1,7,12] the authors integrate the WiFi positioning with the PDR approach using smartphone sensors. In [20] the received signal strength is combined with orientation information obtained by smartphone magnetometer. A positioning method using wireless motes with varying transmission power is described in [28]. In [29] the authors investigate the use of directional antennas for positioning based on RSS and the fingerprinting method. Efficient methods for the generation and updating of fingerprint databases are presented in [30,31,32].

Other sensors used for positioning in indoor environments include Ultrasonic [33], Pseudolites [34,35,36], RFID (including near field communication—NFC) [37,38,39], Ultra Wide Band (UWB) [40,41,42], microphone and light sensor [19]. In [43] an afocal optical flow sensor is introduced for reducing the odometry error in a mobile robot. Camera images [14,15,44] and range data captured by a 2D laser scanner [13] are also used for positioning indoors. In [45] infrared images are used to localize and track moving targets in large indoor environments.

3D mapping and modeling of indoor environments is the focus of several articles in the special issue. In [46] a simultaneous localization and mapping (SLAM) approach based on data captured by a 2D laser scanner and a monocular camera is introduced. In [14,15] visual SLAM using camera images is used for positioning and mapping in indoor environments. The SLAM approach in [47] relies on data acquired by an RGB-D sensor. RGB-D data of indoor scenes are used for detecting small tabletop objects in [48]. In [49,50,51] 3D laser scanning is used for mapping indoor environments. In [50,51] the authors also propose methods for the generation of a building information model (BIM) from the point cloud data.

Applications of indoor location information are discussed in [37,40,52]. In [37] indoor location information is used for tracking health processes and analyzing medical protocols in a hospital. In [40] the authors describe an indoor navigation system for the visually impaired people. In [52] the authors introduce a fading memory model for decision making in indoor emergency situations such as evacuations.

In summary, the special issue presented substantial efforts in the research and development of indoor positioning, tracking and mapping. Many papers address positioning, e.g., by WiFi fingerprinting, but the progress in autonomous approaches for indoor mapping and positioning is also significant. In the presented papers the use of 3D indoor models is relatively limited, but 2D maps are largely applied to support positioning and improve the accuracy. 3D approaches are, however, largely envisaged for simultaneous localization and mapping.

This special issue is the result of the collective efforts of many individuals. We wish to thank the authors for contributing high quality research articles to the special issue. We are also grateful to the anonymous reviewers who thoroughly reviewed the submissions and provided constructive feedback to the authors. We hope the special issue will provide new insights and stimulate further research in indoor mapping, positioning and navigation.
